# Identification of a Novel *NLRP12* Nonsense Mutation (Trp408X) in the Extremely Rare Disease FCAS by Exome Sequencing

**DOI:** 10.1371/journal.pone.0156981

**Published:** 2016-06-17

**Authors:** Xiaoru Xia, Caijun Dai, Xiaochun Zhu, Qiumei Liao, Xu Luo, Yangyang Fu, Liangxing Wang

**Affiliations:** 1 Department of Rheumatology, the First Affiliated Hospital of Wenzhou Medical University, Wenzhou, China; 2 Department of Pneumology, the First Affiliated Hospital of Wenzhou Medical University, Wenzhou, China; 3 Department of Wounds and Burns, the First Affiliated Hospital of Wenzhou Medical University, Wenzhou, China; Virginia Tech University, UNITED STATES

## Abstract

Familial cold autoinflammatory syndrome (FCAS) is an extremely rare autosomal dominant inherited disease. Although there are four genes that have been linked with FCAS, its molecular diagnosis has been challenging in a relatively large proportion of cases. In this study, we aimed to investigate the genetic defect of a recruited FCAS family using exome sequencing followed by in-depth bioinformatics analysis. As a result, a novel heterozygous stop-gain mutation (Trp408X) in *NLRP12* was identified in autosomal dominant inherited FCAS with clinical features of recurrent fever and skin urticaria due to cold conditions. When combined with previous studies, all of the reported mutations were found to have occurred in a highly conserved region in the NACHT domain coding sequence in *NLRP12* exon 3, suggesting that a screening strategy for FCAS should focus on this area of the gene. In conclusion, this study demonstrates the importance of exome sequencing for clinical diagnosis of genetic disorders and provides molecular insight into FCAS treatment and diagnosis.

## Introduction

Cryopyrin-associated periodic syndromes (CAPS) are rare prototypical hereditary autoinflammatory diseases with a prevalence of approximately 1 in 360,000 in France [1] and 1 in 1,000,000 in the USA [2]. According to clinical symptoms, CAPS can be divided into three different subtypes: familial cold autoinflammatory syndrome (FCAS), Muckle-Wells syndrome (MWS), and chronic infantile neurological cutaneous articular syndrome/neonatal onset multisystem autoinflammatory syndrome (CINCA/NOMID). FCAS is the mildest type of CAPS disorder, in which exposure to cold temperatures results in recurrent systemic inflammation involving skin urticaria, recurrent fever and/or joint pain. These symptoms can last much longer in MWS (1–3 days) than in FCAS (12–24h). Sensorineural hearing loss and secondary amyloidosis can be observed in some MWS and CINCA/NOMID patients. In addition to cutaneous involvement, bone deformities, progressive vision loss and central nervous system manifestations, such as aseptic meningitis, can also appear in CINCA/NOMID [3].

The heterogeneity of clinical symptoms suggests that different genes may be responsible for different phenotypes. It is known that FCAS displays autosomal dominant inheritance [4]. Mutations in four genes–*NLRP3*, *NLRC4*, *NLRP12* and *PLCG2*– have been linked to FCAS [5–8]. For example, a number of *NLRP3* mutations have been associated with adult-onset FCAS. Specifically, p.Leu353Pro and p.Leu305Pro in *NLRP3* result in the development of FCAS [9, 10], while the mutations p.Phe309Ser and p.Tyr570Cys tend to result in more severe phenotypes such as neurological disorders [11]. Mutations in *PLCG2* are found in most patients with characteristic cutaneous manifestations [12]. Additionally, a group of patients who exhibit extreme sensitivity to cold were reported to carry the p.D294E mutation in *NLRP12* [13]. However, the genotype-phenotype correlations are still not fully clear due to a limited number of reports on FCAS genetics. At present, the existence of additional genes underlying FCAS is unclear. More detailed knowledge would be helpful for early diagnosis and the development of therapeutic strategies for targeting the mutated gene.

In this study, we aimed to identify the genetic defects in a family diagnosed with FCAS and were especially interested in novel and previously identified disease genes. Exome sequencing is a highly versatile and effective approach for the detection of novel disease-causing genes and mutations. Thus, in this study, we applied exome sequencing to genetic mutation detection in one family affected by FCAS. As a result, a novel probable disease-causing mutation (p.Trp408X) in *NLRP12* was identified in one family affected by autosomal-dominant FCAS, which provided molecular insight into FCAS treatment and diagnosis.

## Materials and Methods

### Patient recruitment

This study conformed to the guidelines of the Declaration of Helsinki and was approved by the Ethics Committee of the First Affiliated Hospital, Wenzhou Medical University. Written informed consent was obtained from all participating patients or their guardians. All of the patients were clinically evaluated by physicians experienced in FCAS diagnosis. Proposed diagnostic criteria for FCAS in these patients suggested an autosomal dominant inheritance pattern. The classical symptoms are recurrent episodes of rash and fever that can be induced by exposure to natural or experimental cold conditions. Symptoms begin during childhood and do not become worse during the patient’s lifetime. The duration of most attacks is less than 24h. Arthralgia and conjunctivitis combined with rash and fever occur in most patients. Deafness, periorbital edema, lymphadenopathy, and serositis are rare in FCAS.

### Sample preparation

A total of 5 ml of peripheral blood were drawn from each patient and control (unaffected family members); the blood was collected in EDTA tubes and stored at -80°C. The individuals described in this study provided written informed consent for publication of case details (as outlined in the PLOS consent form). Genomic DNA was extracted from 1 ml of each blood sample using the DNeasy Blood and Tissue Kit (Qiagen, Germany). DNA quality and quantity were determined using a Nano drop 2000 spectrophotometer (Thermo Scientific). All DNA samples had OD_260_/OD_280_ values of 1.7–2.0 and were not degraded as determined by gel electrophoresis. The sample concentration was ≥50 ng/μl, and for most patients, the quantity was higher than 6 μg. Total DNA was stored at -80°C until use.

### Exome sequencing

For each DNA sample to be sequenced, a single library was prepared using 3 μg of high molecular weight genomic DNA as the starting material using the Agilent SureSelect Library Prep kit. Exome capture was performed using an Agilent SureSelect Human All Exon kit. The samples were then tagged by PCR with different index (barcode) sequences. After assessing the quality and quantity of each library and pooling indexed samples, multiplex sequencing was performed using an Illumina HiSeq 2000 as a 100+100bp paired-end run. We have deposited the raw data on the Figshare (https://dx.doi.org/10.6084/m9.figshare.3406516.v9; DOI: 10.6084/m9.figshare.3406516).

### Identification and annotation of SNPs and InDels

To identify genetic variants, we excluded 3'/5' adapters using Cutadapt implemented in Trim Galore software, and low quality reads were filtered for quality control. Only the reads in which the Phred-scaled sequencing quality was greater than 30 and the read length was greater than 80 bp were used. Each read was mapped to the human reference genome (hg19) using the Burrows-Wheeler Aligner (BWA) program. The GATK (Genome Analysis Toolkit) package was used for InDel realignment, recalibration and variant detection. Duplicated reads were removed using Sequence Alignment/Map tools (SAMtools), and only uniquely mapping reads were used for variation detection. SNP/InDels detected in all affected individuals and absent from unaffected family controls were retained. Detected sequence variants present in the dbSNP, 1000 Genome, ExAC, ESP 6500 and in an in-house Chinese Exome Database (1,500 Chinese Han individuals) were removed. SNVs predicted as deleterious by SIFT (http://sift.bii.a-star.edu.sg/), PolyPhen-2 (http://genetics.bwh.harvard.edu/pph2/), and Mutation Taster (www.mutationtaster.org/) programs were used. Annotation of the variants, such as locations (exonic, intronic, intergenic, etc.) and effects on protein coding sequences (synonymous, missense, nonsense, frameshift, etc.), was performed using an in-house bioinformatics tool with RefSeq (http://www.ncbi.nlm.nih.gov/refseq/) and UCSC gene annotations (hg19, from UCSC).

A total of 100 ng of genomic DNA from each sample was used to validate potential causative genes by conventional PCR and Sanger sequencing. Primers for the detected potential disease-causing mutation Trp408Xin *NLRP12* were 5’-CCTGGAAACTCAAGTGGATG-3’ (forward) and 5’-TGAGGCAGAAAGGAAGGAATA-3’ (reverse).

### Protein structural modeling

The tertiary structures of human wild-type and mutant *NLRP12* were predicted using Rosetta software (https://www.rosettacommons.org/), and the results were saved as a PDB file format. The PDB files obtained for the two samples were then used by PyMOL (http://pymol.org/) to visualize the structures of these proteins.

## Results

### Characteristics of the patient cohort

**Fig 1:**
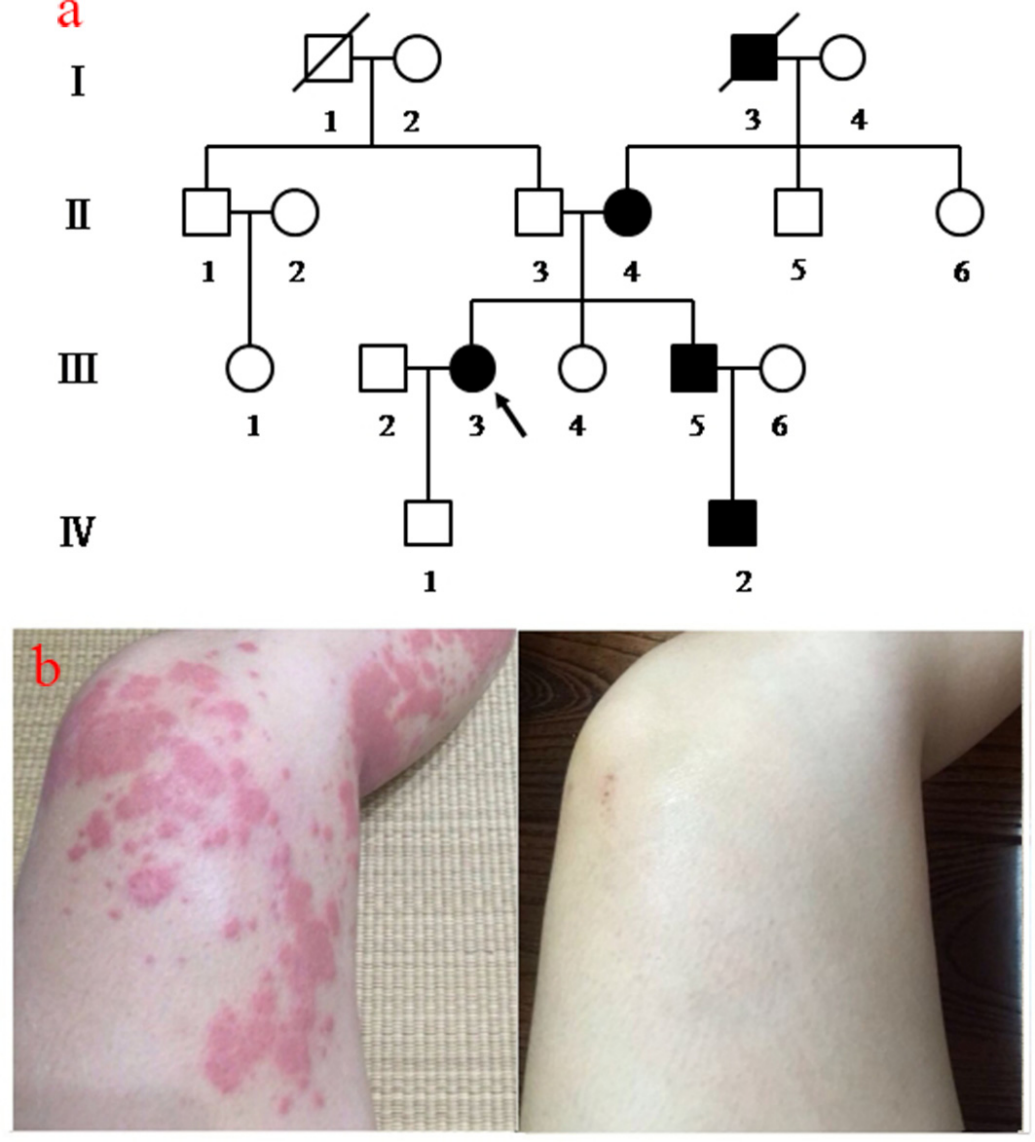
FCAS Pedigree and Photographs of Rashes. (a) FCAS pedigree and analysis. Squares and circles indicate males and females, respectively; black symbols and unfilled symbols indicate affected and unaffected individuals, respectively. The proband is indicated by an arrow. (b) Photographs of cold urticaria provoked after exposure to a 4°C outdoor atmosphere for 30 minutes and after being at room temperature with no rashes.

The patient cohort for this study was a family composed of 18 individuals, as shown in Fig 1A. Based on the pedigrees, the inheritance pattern of the FCAS family in this study was most likely autosomal dominant. None of the patients had a history of asthma and all experienced urticarial rashes during childhood before 1 year of age (Fig 1B). The symptomatic periods in these patients were short and lasted for 12–24 h, and appeared 1 or 2 h after exposure to cold conditions. The urticarial-like rashes appeared on the limbs and the trunk and were sometimes accompanied by fever, with most patients experiencing joint pain. These symptoms disappeared without treatment in this affected family. Hearing loss, mental retardation and renal disturbance were not observed in any of the affected individuals in this family, nor was there any incidence of systemic disorders. The clinical data are summarized in Table 1. A cold-induced dermatographism test result was negative. Elevated erythrocyte sedimentation rates and C-reactive protein levels were observed in all four living patients. Patients tested negative for autoantibodies. Based on these clinical manifestations, the most probable diagnosis in this family was FCAS.

**Table 1:** Clinical Summary of the Individuals in this Study. M, Male; F, Female; +, Affected; -, Not affected.

Individual ID	Gender	Age (Y)	Ageat Onset (M)	Cold-induced urticarial rash	Fever	Arthralgia	Conjunctivitis
**II:4**	F	65	9	+	+	-	-
**III:3**	F	35	5	+	+	+	-
**III:5**	M	37	6	+	+	+	-
**IV:2**	M	10	7	+	+	-	-

### Mutation analysis

**Fig 2:**
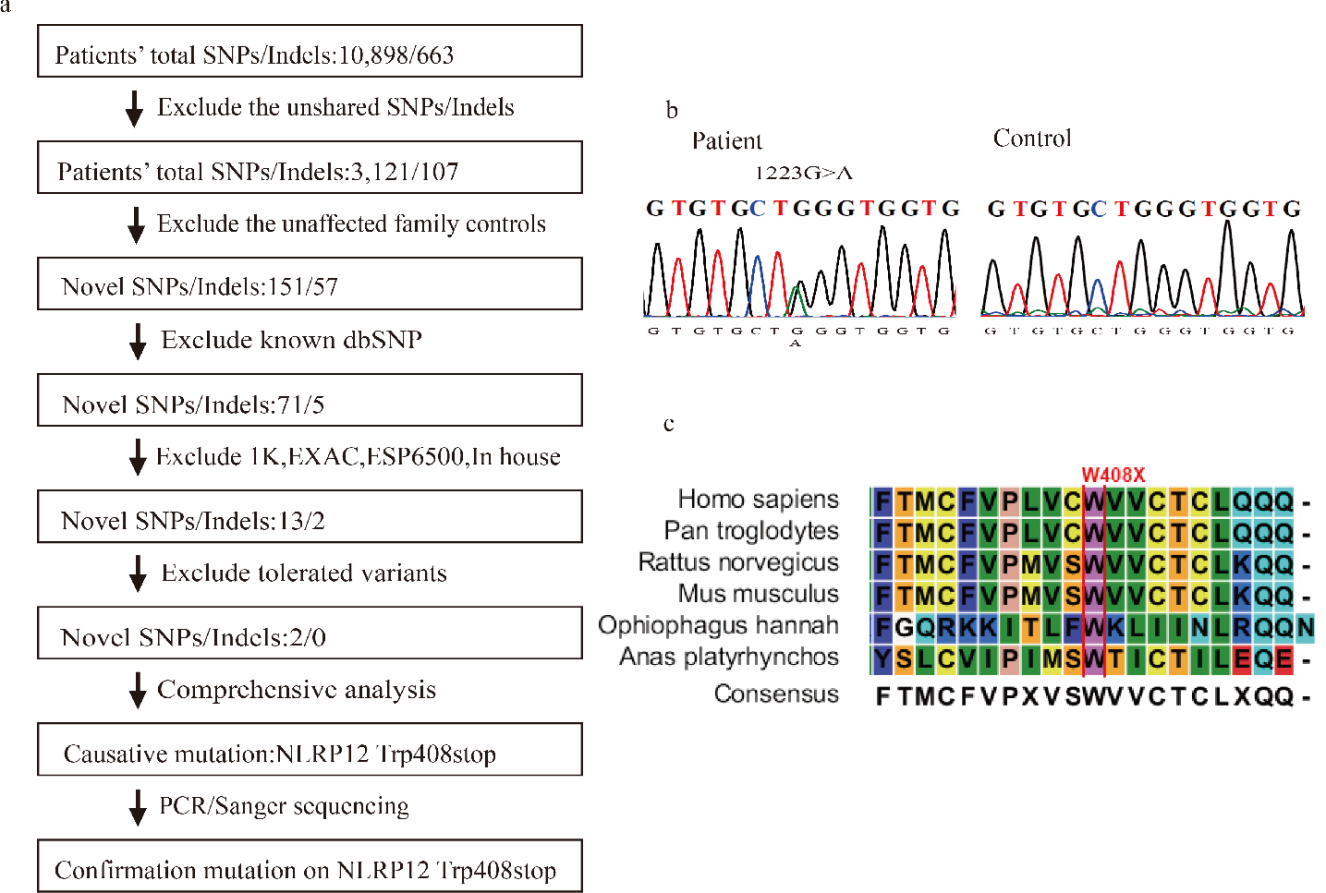
A c.1223G>A Mutation in *NLRP12* Identified in an FCAS Family. (a) Flow diagram of the filtering analysis of the exome sequencing data. The data from the patients and controls were compared, and known SNPs and InDels were excluded as described in the Materials and Methods section. The causative mutation was identified after a comprehensive analysis. (b) The c.1223G>A mutation in patient III-5 compared to the control was confirmed by Sanger sequencing. The position is indicated by an arrow. (c) Sequence alignment of multiple species. The red line shows the position of the p.Trp408X mutation.

Due to rapidly decreasing costs, exome sequencing has been increasingly applied as a powerful clinical diagnostic tool [14]. It can rapidly provide information that can be used for guidance and evidence relating to the causes of many diseases. In our study, to identify potential causative mutations for FCAS, we utilized whole exome sequencing (WES) to analyze three affected individuals (II-4, III-3 and IV-2) and three unaffected family members (II-5, III-4 and IV-1) of one family diagnosed with FCAS (Fig 2A). Here, we adopted similar strategies to those used in other published works for WES sequencing data processing and analysis [15–17]. After removing the 3’ and 5’ adapters using Cutadapt and the poor quality reads by FastQC, an average of 5.90 Gb of clean data remained per exome (Table 2). Next, we used BWA to map these clean reads to the human reference genome (hg19), allowing two mismatches per read. Overall, 99.13% of the reads were aligned to the reference genome. Among these mapped reads, approximately 55.33% were located in target regions with a mean of 59.48x sequencing depth. We observed that 96.03%, 92.37% and 83.43% of the sites in the target regions had greater than 4x, 10x and 20x read coverage, respectively. Above all, these data fully reflect the reliability of our sequencing data and provide a strong basis for follow-up analysis.

**Table 2:** Summary of the Exome Sequencing Data.

Sample	Clean_data (Gb)	Aligned (%)	Initial bases on target (Mb)	Base covered on target (Mb)	Effective bases on target (%)	Average sequencing depth	10× coverage (%)	10× coverage (%)	20× coverage (%)
**II-4**	5.43	99.20	50.34	49.08	57.00	55.39	95.70	91.80	82.70
**II-5**	5.19	99.70	50.34	49.05	60.00	56.51	95.80	92.00	83.10
**III-3**	6.24	98.70	50.34	49.47	49.30	58.85	96.70	93.50	84.70
**III-4**	6.58	99.10	50.34	49.13	55.40	64.64	96.20	93.00	85.00
**IV-1**	6.86	98.40	50.34	49.02	57.40	69.82	95.60	91.80	83.80
**IV-2**	5.09	99.70	50.34	49.21	52.90	51.65	96.20	92.10	81.30
**Mean**	5.90	99.13	50.34	49.16	55.33	59.48	96.03	92.37	83.43
**STDEV**	0.69	0.48	0.00	0.15	3.44	6.07	0.38	0.65	1.25

Using GATK, we identified 10,898 coding single nucleotide variants (SNVs, in this case, consisting of missense, stop-loss, stop-gain and splice site mutations) and 663 short coding insertions or deletions (InDels, including frameshift and non-frameshift mutations) by sequencing three patients in total (Fig 2B, Table 2). According to the autosomal dominant inheritance pattern, we assumed that the pathogenic cause of the disease in this family was due to the same heterozygous mutation in one gene. Therefore, we excluded the variants that were not shared in all affected individuals. After this exclusion, 3,212 SNVs and 107 InDels remained. To identify the causative mutation, the variants present in three normal family members were filtered, following which, the shared SNVs and InDels were reduced to 151 and 57, respectively. Then, we excluded the variants in common databases of normal controls, including dbSNP v138 (http://www.ncbi.nlm.nih.gov/projects/SNP/), 1000 Genomes (http://www.1000genomes.org/), ExAC (http://exac.broadinstitute.org/), ESP6500 (http://evs.gs.washington.edu/EVS/) and our 1,500 Chinese in-house exome data sequenced by Beijing Genomics Institute, Shenzhen, China.

The number of remaining SNVs and InDels were 13 and 2 (S1 Table), respectively. These mutations were further analyzed with computational tools to predict their damaging effects. InDels were considered pathogenic if they caused a frameshift. For the nonsynonymous SNV mutations, we predicted the damaging effect using three tools [18], including PolyPhen-2, SIFT and Mutation Taster. After this step, two potentially damaging SNVs remained. We then performed a more comprehensive analysis of gene function, gene expression and intersection with known Mendelian disease loci. In addition, we reviewed these genes in the context of relevant literature about this disease. Finally, we identified a previously unreported heterozygous stop-gain mutation (Trp408X) in *NLRP12*. The other candidate mutation (PERM1, c.G1661A, p.R554Q) was not followed up because it was functionally unrelated to FCAS.

We conducted PCR and Sanger sequencing on all six family members and confirmed the heterozygous stop-gain (nonsense) mutation in the three affected subjects but not in the normal subjects (Fig 2C). Subsequently, we performed PCR and Sanger sequencing on the other members (all normal subjects) of the pedigree with available DNA samples and confirmed that they did not have the Trp408X mutation in *NLRP12*. This finding demonstrates the practical applicability of WES.

### Protein structural modeling

**Fig 3:**
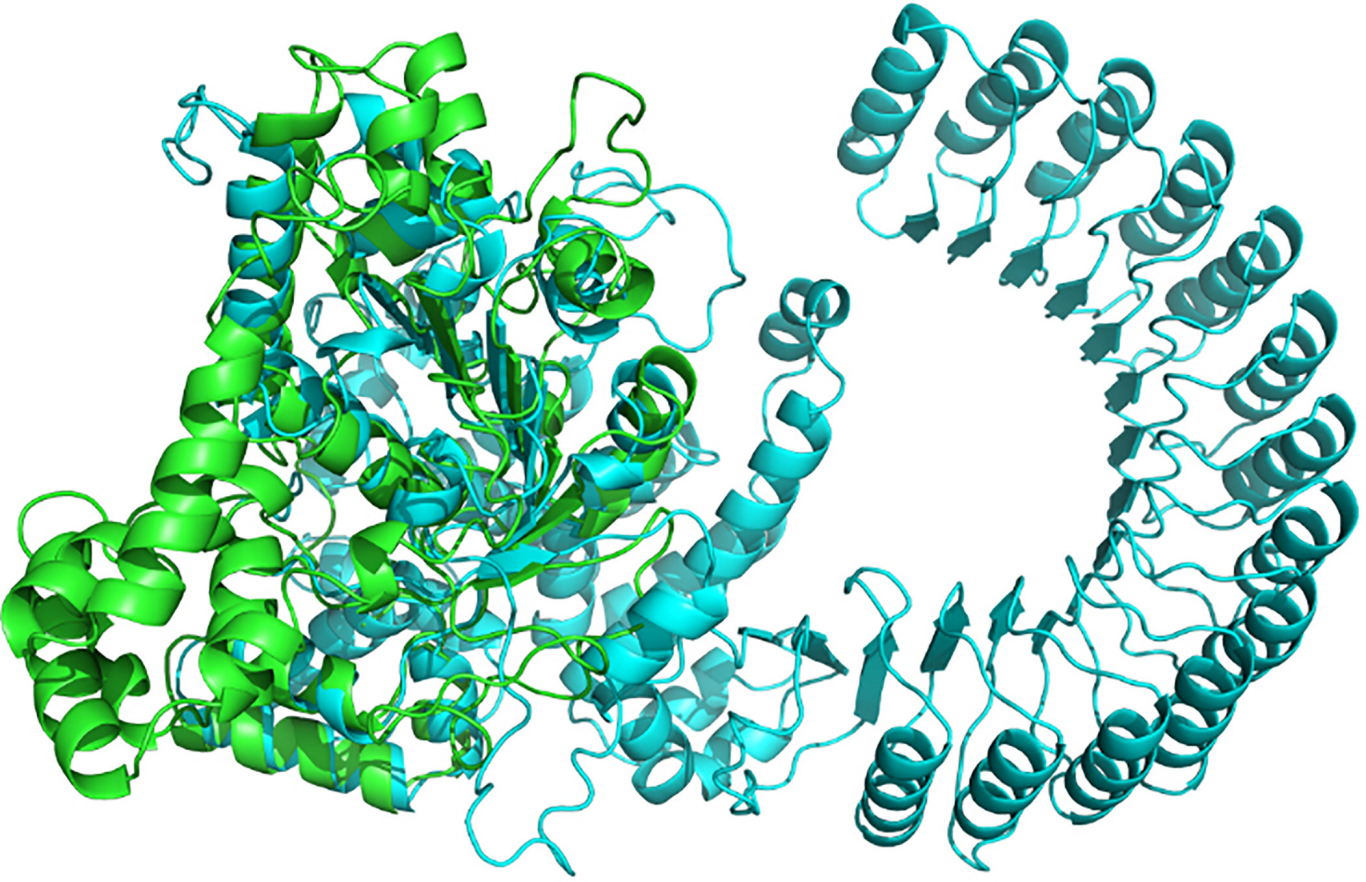
Structural Difference between Wild-type and C-terminal Truncated *NLRP12* Proteins. Function predictions of the truncated protein and the wild-type (full-length) *NLRP12* were carried out. The truncated protein (p.Trp408X) is represented in green. The full length protein is represented in blue. It is clear that the mutation disrupts the integrity of the *NLRP12* protein.

The *NLRP12* protein (http://www.uniprot.org/uniprot/P59046, ENSP00000319377) consists of an N-terminal DAPIN domain, a NACHT domain and C-terminal LRR (leucine-rich) repeats. The DAPIN/Pyrin domain is located between amino acids 1–95, and the NACHT domain is located at residues 211–528, with the remaining part of *NLRP12* comprising the C-terminal LRRs (828–1,049). Using Rosetta software (https://www.rosettacommons.org/software), we predicted the 3D structure of the wild-type *NLRP12* protein (1–1049) and mapped out the Trp408 residue in the structure (Fig 3). The p.Trp408X mutation results in a protein product with a deletion of the entire C-terminal LRR region and partial deletion of the NACHT domain. This nonsense mutation at this key region may lead to protein dysfunction and therefore contribute to disease pathogenesis.

## Discussion

FCAS is a dominantly inherited disease with the recurrent symptoms of early onset and lifelong urticarial-like rashes after exposure to cold environments. Clinical symptoms are mild and include rashes, polyarthragia and fever. These symptoms occasionally overlap with symptoms of CAPS, MWS and CINCA/NOMID [13]. FCAS is an extremely rare syndrome and is not a well-recognized disease. Therefore, we speculate that many more cases may still be undiagnosed or even misdiagnosed. The familial history of this disease indicates that genetic factors may play an important role in disease development.

FCAS has shown distinct genetic heterogeneity, which suggests that the disease may be caused by mutations in more than one gene. Therefore, we sought to investigate whether additional disease genes besides those previously reported could lead to FCAS. WES has shown great promise as a powerful tool for understanding the genetic architecture of human disease as well as in diagnostic procedures. Through exome sequencing techniques, the underlying genetic cause of some diseases has been discovered [14, 19, 20]. In addition, with the rapid development of next-generation sequencing techniques, the cost of exome sequencing has drastically decreased. Therefore, in this study, we directly employed exome sequencing to investigate the inherited genetic defect in a FCAS family. One reason we did not directly use PCR and Sanger sequencing is that it is expensive to perform these procedures on all four known FCAS genes. Another reason is that it is often difficult and time-consuming to amplify all the exonic regions using PCR. Our results demonstrated that exome sequencing can replace PCR and Sanger sequencing and can be used as a genetic diagnostic tool for larger pedigrees in the future.

In this study, we employed a commonly used protocol for exome sequencing data analysis. First, BWA and GATK software were used for the detection of mutations from sequencing data. Second, we took advantage of in-house exome databases and several public databases as normal controls. In our study, we used this protocol to successfully identify a pathogenic mutation in a family with FCAS, which illustrated the effectiveness of exome sequencing technology for the identification of disease-causing genes for an extremely rare disease. We acknowledge one report that used exome sequencing to identify the mutation in *NLRP12* for common variable immunodeficiency disease [21].

According to the literature, *NLRP12* has six causative mutations linked to FCAS, including the present mutation. These mutations and their corresponding clinical characteristics are summarized in Table 3 [13, 21–26]. Surprisingly, we found that all of the reported mutations were located in exon 3 and intron 3 (the flanking sequence of exon 3) of *NLRP12*. This finding indicates that this region of *NLRP12* may be a mutational hotspot in FCAS and highlights that more attention should be paid to this region when screening for mutations in FCAS. Furthermore, we provide insights into the clinically heterogeneous nature of FCAS because, even when mutations are encoded within the same exon, the clinical manifestations are dramatically different (Table 3). Specifically, episodic fever, arthralgia, myalgia, and urticaria could be triggered by cold exposure in individuals carrying the p.R284X mutation in *NLRP12*. Recurrent bouts of fever and severe fatigue (musculo-skeletal symptoms) have been observed in patients carrying the p.F402L mutation in *NLRP12*. Notably, the p.R284X mutation in *NLRP12* may be linked to sensorineural hearing loss.

**Table 3:** NLRP12 Gene Mutations and Diseases. ND, Not detectable; NA, No answer.

Mutation	Location	Phenotype	Autosomal dominant	Esp6500 Allele freq	1000G Allele freq	ExACAllele freq	References
**c.850C>T, p.R284X**	Exon3	Onset at first days of life; Episodic fever, arthralgia, myalgia, and urticaria by cold; sensorineural hearing loss.	Yes	7.690 e-04	2.396 e-03	2.277e-04	[7, 27]
**c.882C>G, p.D294E**	Exon3	Onset at childhood; Cold-induced urticarial rash, arthralgias and myalgia; Elevation of acute-phase reactants	Yes	ND	ND	8.132e-06	[13]
**c.910C>T, p.H304Y**	Exon3	Classical FCAS2 clinical manifestation	NA	2.691 e-03	2.196 e-03	4.220e-03	[26]
**c.1223G>A p.W408X**	Exon3	Onset at first year of life Episodic fever, urticaria and arthraigia triggered by cold	Yes	ND	ND	ND	In this study
**c.1206C>G, p.F402L**	Exon3	Recurrent fever, skin rashes, abdominal pain and tonsillitis; Good response to IL-1 inhibitor;	Yes/NA	5.113 e-02	2.696 e-02	5.200 e-02	[22, 23]
**c.2072+3insT, p.Val635ThrfsX12**	Intron3	Onset at first year; Episodic fever, abdominal pain and arthralgia triggered by cold.	Yes	ND	ND	ND	[7]

**Fig 4:**
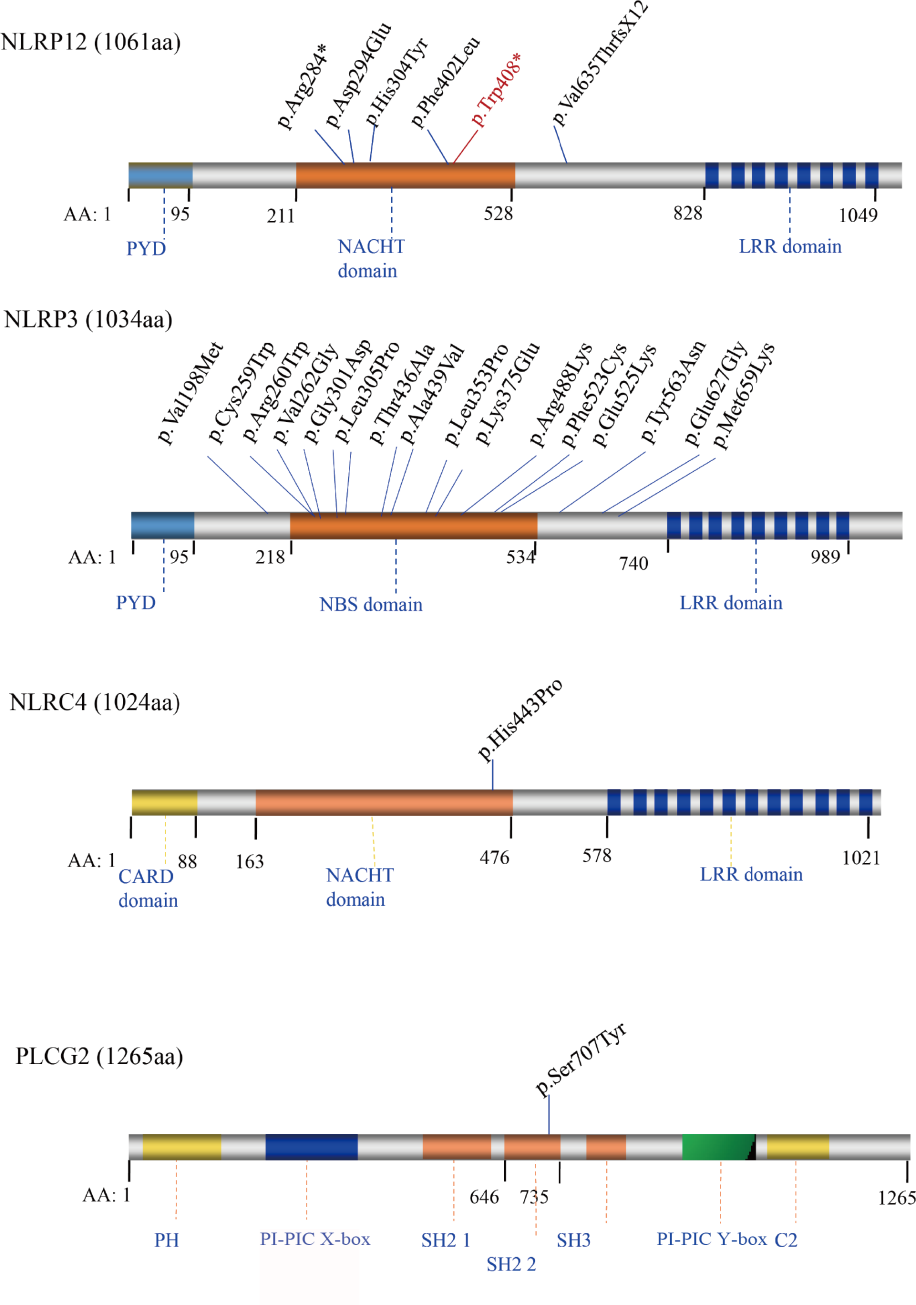
Schematic Diagram of Mutations in the Encoded Proteins of *NLRP12/NLRP3/NLRC4/PLCG2* Genes Associated with FCAS. Rectangles with different colors indicate specific protein domains. The solid blue line indicates the position of the reported mutation, and the solid red line shows the p.Trp408* mutation related to our study.

We also mapped all the reported mutations in *NLRP12*, *NLRP3*, *NLRC4* and *PLCG2* (Fig 4), which are the four genes linked with FCAS. With the exception of *PLCG2*, almost all of the reported mutations were located at a highly conserved region in the NACHT domain, which demonstrates that this domain is the key functional region of the *NLRP* gene family in the context of FCAS. As such, NACHT is a key region in the clinical molecular diagnosis of FCAS and should be prioritized for sequencing. The NACHT domain is closely related to the nucleotide-binding adaptor NB-ARC, which is shared by apoptotic ATPases, such as the apoptotic protease activating factor-1 (APAF1), NLR family CARD domain-containing proteins (NLRC4), plant resistance (R), and Caenorhabditis elegans cell death (CED-4) proteins [28]. Proteins containing these domains are considered to be regulators of programmed cell death in animals and plants via an ATP-dependent protein oligomerization process. To date, no NACHT domain crystal structure has been published for any NLRP family member. Although the contributions of the NACHT domain to the specific biological functions of the various *NLRP* genes are unknown, it is proposed that the central NACHT nucleotide-binding domain of the *NLRP* family offers a novel opportunity for pharmacologic intervention [28].

*NLRP12* encodes a member of the CATERPILLER family of cytoplasmic proteins, which function as important components of inflammation and immunity [29]. Human *NLRP12* is expressed by monocytes/macrophages and granulocytes and is a regulator of NF-κB activation, where it acts as a negative controller of inflammation, suppressing both canonical and non-canonical NF-κB activation [27, 30]. In the absence or mutation of *NLRP12*, biosynthesis of cytokines including IL-1 is increased. The weak clinical symptoms of FCAS are, therefore, likely due to the accelerated kinetics of IL-1 secretion in the absence of an overall increased amount of the secreted cytokine [13]. The molecular consequences of several mutations have been studied. *In vitro* functional expression studies have shown that the mutations (p.R284X and 2072+3insT) in *NLRP12*, which have a similar phenotype in patients (Table 3), had reduced inhibitory action against NF-κB compared to wild-type *NLRP12*. A dominant mode of expression through haploinsufficiency was suggested [7]. We speculate that a similar molecular consequence may be present, as observed with the p.Trp408X mutation. Further studies are required to elucidate the detailed molecular consequences of this mutation.

To the best of our knowledge, this is the first case to report a novel disease-causing mutation (p.Trp408X) in *NLRP12* for autosomally dominant inherited FCAS. We suggest that the screening strategy for FCAS should be targeted to gene fragments coding for the key region (the NACHT domain encoded by exon 3). In addition, this study provides further molecular insight for FCAS treatment and diagnosis, as well as illustrating the important role of exome sequencing in the clinical diagnosis of genetic disorders.

## Supporting Information

S1 Table The list of 13 SNVs and 2 Indels corresponding to Fig 2A. (DOCX)
