# Supplementary material for: Identification of a Novel NLRP12 Nonsense Mutation (Trp408X) in the Extremely Rare Disease FCAS by Exome Sequencing
Source: PLoS One. 2016 Jun 17;11(6):e0156981. doi: 10.1371/journal.pone.0156981 (PMC4912109; doi:10.1371/journal.pone.0156981)
Supplement: S1 Table — (DOCX) [file pone.0156981.s001.docx]

**S1 Table. The list of 13 SNVs and 2 Indels corresponding the Fig 2a.**

| Gene | Nucleotide change | Aminoacid change | Polyphen-2 | SIFT | Mutation Taster |
| --- | --- | --- | --- | --- | --- |
| PERM1 | c.G1661A, | p.R554Q | Probably_damaging | Damaging | Disease_causing |
| NLRP12 | c.C1223T | Trp408X | Probably_damaging | Damaging | Disease_causing |
| PHKA1 | c.T242C | p.V81A | Possibly_damaging | Tolerable | Disease_causing |
| KIR3DL1 | c.G883A | p.G295S | Probably_damaging | Damaging | Polymorphism |
| ZNF717 | c.C1505T | p.T502I | Probably_damaging | Damaging | Polymorphism |
| ZZEF1 | c.G8795T | p.C2932F | Possibly_damaging | Tolerable | Disease_causing |
| CDH15 | c.T58G | p.L20V | Benign | Tolerable | Polymorphism |
| COL12A1 | c.T500C | p.V167A | Benign | Tolerable | Disease_causing |
| TAAR5 | c.A241G | p.M81V | Benign | Damaging | Polymorphism |
| KIR2DL1 | c.T986C | p.I329T | Benign | Tolerable | Polymorphism |
| PDS5B | c.C3964A | p.P1322T | Benign | Tolerable | Polymorphism |
| TAS2R30 | c.C193A | p.H65N | Benign | Tolerable | Polymorphism |
| UGT1A7 | c.T657A | p.F219L | Benign | Tolerable | Polymorphism |
| TCHH | c.1517_1518insGGAGAGGCGCGAGCAGCA | p.Q506delinsQERREQQ | - | - | - |
| RPTN | c.474_509del | p.158_170del | - | - | - |
